# Can Physical Exercise Promote the Development of Teenagers’ Non-Cognitive Ability?—Evidence from China Education Panel Survey (2014–2015)

**DOI:** 10.3390/children9091283

**Published:** 2022-08-25

**Authors:** Shiwei Yuan, Qian Gu, Yuanyuan Lei, Jinbo Shen, Qian Niu

**Affiliations:** 1 Wuxi Higher Health Vocational Technology School, Wuxi 214000, China; 2 School of Physical Education, Shandong University, Jinan 250100, China; 3 Physical Education College, Hebei Normal University, Shijiazhuang 050000, China; 4 Taiyuan Institute of Technology, Taiyuan 030008, China

**Keywords:** social behavior, communication ability, perseverance, emotional control, school adaption, creative thinking, educational expectation, adolescent

## Abstract

Background: With the development of educational philosophy, physical exercise is considered to be an important factor in the development of individual cognitive abilities in adolescents. However, the effects of physical exercise on non-cognitive abilities in adolescents remain inadequate. Objective: This study examined the effect of physical exercise on non-cognitive ability and further examined the differences in this effect in different groups. Methods: Survey data on non-cognitive ability, physical exercise and covariates were collected in the China Education Panel Survey (2014–2015) from a nationally representative sample of adolescents (*n* = 7904) in the eighth (follow-up) grade. Results: The results show that, after controlling for the influence of other factors, physical exercise has a promoting effect on six non-cognitive abilities—social behavior (*p* < 0.001), communication ability (*p* < 0.01), perseverance (*p* < 0.05), school adaptation (*p* < 0.001), educational expectation (*p* < 0.01) and creative thinking (*p* < 0.01)—and there is no significant effect on emotional control (*p* > 0.05). Furthermore, the development of non-cognitive ability in physical exercise shows certain group differences. Conclusions: There are gender differences in the performance of non-cognitive ability. Girls perform better than boys in non-cognitive ability. Physical exercise is an important means to develop non-cognitive ability, which indicates that non-cognitive ability has plasticity in adolescence. Future intervention may improve the non-cognitive ability of Chinese adolescents by increasing physical exercise.

## 1. Introduction

Cognitive skills and non-cognitive skills play an important role in promoting well-being throughout life [1,2]. In particular, cognitive skills have been identified as important predictors of adolescent outcomes such as test scores [3,4], educational achievement [5,6], labor market outcomes [7,8] and health [9]. However, with the deepening of research, individuals have begun to realize that cognitive ability may be one (but not the only) factor affecting academic performance and human capital. Recent studies have shown that in the rate of return to education, when the effects of cognitive ability, family background and some demographic variables are controlled, there is still two-thirds of the variance that cannot be explained [10]. At the same time, the relationship between non-cognitive ability and personal income and academic performance has been supported by increasing empirical evidence [11,12]. The importance of non-cognitive ability has begun to attract further attention. Non-cognitive ability, also known as personality traits, usually refers to the persistent pattern of an individual’s thoughts, feelings and behaviors—namely, the response mode of fixed tendencies and trends that always appear in an individual in a specific environment and situation [13].

The Big Five Personality Theory is an important theoretical framework for measuring non-cognitive ability and has been widely used. It includes five dimensions: openness, conscientiousness, extroversion, agreeableness and neuroticism [14]. At the same time, sociologists have a rich understanding of its connotations, such as leadership, perseverance, self-esteem, internal and external control points, other psychological factors, educational expectations, school-related attitudes and behaviors, all related to the individual’s academic and future achievements [15,16]. On this basis, combined with the questionnaire content in the China Education Panel Survey (2014–2015), this paper determines seven non-cognitive ability indicators. Among them, creative thinking (openness), perseverance (conscientiousness), communication ability (extroversion), school adaptation (agreeableness) and emotional control (neuroticism) are the proxy indicators of the Big Five Personality Theory. At the same time, educational expectation and social behavior are taken as supplementary indicators of the Big Five Personality Theory. All the above indicators constitute the seven non-cognitive abilities studied in this paper.

Studies have shown that non-cognitive ability possesses plasticity [17]. Among many factors affecting the non-cognitive ability of teenagers, the importance of physical exercise is self-evident [18]. In the growth process of teenagers, physical exercise not only improves their physical fitness, but also improves their ability to adapt to society, such as self-expression, mutual cooperation and emotional control [19]. However, other studies have found that the improvement of adolescents’ non-cognitive ability can reverse the promotion of physical exercise behavior. Combined with the Theory of Planned Behavior (TPB), Courneya et al. found that conscientiousness and extroversion personality traits had a positive effect on physical exercise behavior, while neuroticism had a negative effect on physical exercise behavior [20]. Bowman found that extraversion, emotional stability and other personality traits were positively correlated with physical exercise motivation, while openness was negatively correlated with physical exercise motivation [21]. Wilson and Dishman [22] pointed out through a meta-analysis that non-cognitive ability was positively correlated with physical exercise behavior at a low degree.

As a type of ability closely related to personal future achievements, the question of how to cultivate non-cognitive ability in the adolescent stage has become a significant problem in the field of education. There have been a great deal of research results on the relationship between physical exercise and non-cognitive similarity indexes, but only a few studies specifically on junior middle school adolescents [23,24]. In the research process, simple sampling with a small sample is generally adopted, and its representativeness is questionable. In the selection of control variables, only general demographic variables (such as gender, age, residence type, etc.) are controlled, and non-physical exercise factors affecting these non-cognitive abilities are not controlled (such as parent–child relationships, cognitive ability, academic score, etc.) [11,25,26]. Therefore, this study suggests that physical exercise may play an important role in the development of adolescents’ non-cognitive abilities. Moreover, few studies have examined the relationship between physical exercise and adolescents’ non-cognitive abilities on a national scale. Therefore, this study will explore the influence of physical exercise on the development of adolescents’ non-cognitive abilities by using the survey data of the China Education Panel Survey (CEPS). We can further understand the group differences in the impact of physical exercise on non-cognitive ability by exploring the effects of gender, household registration type, one-child status, boarding and family economy on physical exercise and non-cognitive ability. This study provides some references for educational reform policies to cultivate students’ cognitive and non-cognitive abilities. At the same time, the empirical evidence of the impact of physical exercise on adolescent non-cognitive development is supplemented.

## 2. Materials and Methods

### 2.1. Individuals

The China Education Panel Survey (CEPS) is a nationally representative, large-scale follow-up survey project for junior high school students, their parents, teachers and school leaders. In the baseline survey, around 20,000 students from 112 schools and 438 classes were selected for the survey. The second survey successfully tracked 10,000 students from junior high school (currently in grade 8); we applied for the data of CEPS (2014–2015) in February 2019 and obtained approval online.

According to the purpose of this study, we formulated the inclusion criteria for the study subjects: demographic data such as gender, household registration type, etc., non-cognitive ability data, physical exercise data and control variable data. Exclusion criteria: samples with missing values in physical exercise, gender, household registration type, only child status, boarding status and family economy were deleted. According to the inclusion criteria and study needs, we sorted out the CEPS (2014–2015) data, supplemented some variables through the baseline survey data and obtained a total of 7904 survey samples. In all samples, the average age was 14.1 ± 0.6 years (including 14.2 ± 0.7 years for boys and 14.1 ± 0.6 years for girls). The mean BMI was 18.5 ± 3.4 (18.9 ± 3.8 for boys and 18.0 ± 3.0 for girls).

### 2.2. Non-Cognitive Ability and Data Synthesis

The non-cognitive abilities considered in this paper include social behavior, communication ability, perseverance, school adaptation, educational expectation, emotion control and creative thinking.

#### 2.2.1. Social Behavior

Social behavior refers to the degree to which individuals comply with social norms. It is obtained by dividing the average score of the prosocial behavior scale by the average score of the antisocial behavior scale. The higher the scores are, the better individual’s social behaviors perform. The items of the prosocial behavior scale include “helping elders”, “following orders and lining up” and “being nice and honest”. The items of the antisocial behavior scale include “having a fight with others”, “bullying the weak”, “skipping classes, being absent, or truanting”, “copying homework from others, or cheating in exams”, “smoking, or drinking alcohol”, and “going to net bars or video arcades”. The response options are never = 1, seldom = 2, sometimes = 3, often = 4, always = 5.

#### 2.2.2. Communication Ability

Communication ability refers to the extent to which individuals show willingness to communicate, actively participate in communication and show effective and appropriate communication behaviors in the process of interpersonal communication, so as to achieve harmonious relationships between themselves and others [27]. The items examining communication ability include “there are some adults I respect and admire”, “I can chat with adults easily” and “I would apologize if I hurt others unintentionally”. The response options are as follows: strongly disagree = 1, somewhat disagree = 2, somewhat agree = 3, strongly agree = 4.

#### 2.2.3. Perseverance

Perseverance is an individual’s persistence and enthusiasm for their long-term goals. People with a high level of perseverance will continue to work hard and maintain continuous interests even in periods of failure, dilemma or stagnation when completing challenging tasks [28]. The items examining perseverance include “I would try my best to go to school even if I was not feeling very well or I had other reasons to stay at home”, “I would try my best to finish even the homework I dislike” and “I would try my best to finish my homework, even if it would take me quite a long time”. The response options are as follows: strongly disagree = 1, somewhat disagree = 2, somewhat agree = 3, and strongly agree = 4.

#### 2.2.4. Emotional Control

Emotional control refers to the ability to effectively regulate negative emotions such as anger and worry, which is specifically manifested in the ability to better control one’s emotions when encountering setbacks [29]. The items examining emotion control include “feeling blue”, “too depressed to focus on anything”, “unhappy”, “not enjoying life”, “having no passion to do anything”, “sad, sorrowful”, “nervous”, “excessive worry” and “feeling something bad will happen”. The response options are as follows: never = 1, seldom = 2, sometimes = 3, often = 4, always = 5.

#### 2.2.5. Creative Thinking

Creative thinking refers to thinking that can produce novel thinking results. The items examining creative thinking include “able to express himself/herself clearly”, “able to give quick responses”, “a faster learner” and “curious about new stuff”. The response options are as follows: not fit at all = 1, somewhat not fit = 2, somewhat fit = 3, exactly fit = 4.

#### 2.2.6. School Adaptation

School adaptation refers to the ability of students to integrate into the collective life of the class. The items examining school adaption include “most of my classmates are nice to me”, “I often take part in school/class activities” and “I feel close to people in this school”. The response options are as follows: strongly disagree = 1, somewhat disagree = 2, somewhat agree = 3, strongly agree = 4.

#### 2.2.7. Education Expectation

Education expectation is measured by the item “how far do you want to read your books?”, and the expected education stage is transformed into the number of years of education.

The related topics of communication ability, perseverance, school adaptation, emotion control and creative thinking are all processed through principal component factor analysis, and the value of 0–100 is generated through standardized processing. The larger the scores of communication ability, perseverance, school adaptation and creative thinking, the higher the corresponding non-cognitive ability. The larger the score of emotional control, the weaker the capacity for emotional control appears.

$$Y=\left[\left(X-X_{MIN}\right)/\left(X_{MAX}-X_{min}\right)\right]\;\times \;100\;(0–1\;\mathrm{standardized}\;\mathrm{formula}).$$

### 2.3. Physical Exercise and Data Synthesis

In CEPS (2014–2015), the measurement of physical exercise includes two questions, “the number of days of physical exercise per week” and “how many minutes each time”. According to the research needs, we converted it into daily physical exercise time.

### 2.4. Statistical Analysis

All the data in this paper were obtained from the CEPS database in DTA format and analyzed by using STATA/SE15.1. A three-stage analysis was performed based on the study content outlined.

Firstly, we formulated a preliminary description of the characteristics of the data. The format of categorical variables is *n* (%), and continuous variables are represented as mean (M) ± standard deviation (SD). Chi-square tests (for categorical variables) and *t*-tests (for continuous variables) were conducted to test for any gender difference.

Secondly, in exploring the regression analysis of physical exercise on non-cognitive ability, the multiple linear regression method was used, and the results were expressed as β value, *p* value (* *p* < 0.05, ** *p* < 0.01, *** *p* < 0.001) and standard error. We tested whether there was a high correlation between the explanatory variables by calculating the variance inflation factor (VIF). The calculation results of VIF were between [1.031, 1.903], and the average value was 1.276. It can be inferred that there was no multicollinearity between the explanatory variables. In order to further test the quality of the prediction equation, we analyzed the residual of the prediction equation. The skewness and kurtosis tests of normal distribution showed that the skewness and kurtosis of the seven regression equations were all 0, and the residuals met the requirements of normal distribution.

Finally, by multiplying physical exercise with gender, household registration type, only-child status, boarding status and family economy, we set interactive items for moderation analysis. We obtained the group difference in the influence of physical exercise on non-cognitive ability. The results are expressed as β values and *p* values (* *p* < 0.05, ** *p* < 0.01, *** *p* < 0.001).

## 3. Results

Table 1 presents the descriptive statistics of all variables in this paper. A total of 7904 participants were junior high school students, including 4030 boys and 3874 girls. Among the seven non-cognitive abilities studied in this paper, there are significant differences between boys and girls in social behavior, communication ability, perseverance, school adaptation and educational expectation. Girls’ scores are significantly higher than boys’ (*p* < 0.001), while there is no significant difference between boys and girls in creative thinking (*p* > 0.05), and boys’ emotion control ability is significantly better than girls’ (*p* < 0.001) (the larger the score of emotional control is, the weaker the ability of emotional control appears). There is no significant difference between boys and girls in terms of physical exercise time, the core explanatory variable of this paper (*p* > 0.05). In terms of control variables, girls are significantly better than boys in cognitive ability and academic performance. The peer groups around girls are more progressive than those around boys (*p* < 0.001). Teachers pay more attention to girls than boys (*p* < 0.001). Girls’ family education capital is also slightly better than boys’ (*p* < 0.01).

Table 2 shows the regression results of physical exercise on seven non-cognitive abilities: social behavior, communication ability, perseverance, school adaptation, educational expectation, emotional control and creative thinking. After controlling for the influence of gender, household registration type and other variables related to non-cognitive ability, when the degree of participation in physical exercise is higher, the social behaviors are more standardized (*p* < 0.001), the communication ability is stronger (*p* < 0.01), the school adaptation is better (*p* < 0.001), the level of perseverance is higher (*p* < 0.05), the educational expectation is higher (*p* < 0.01), and the creative thinking is better (*p* < 0.01). The results indicate that physical exercise is an important path to develop the non-cognitive ability of junior middle school students.

Table 3 shows the impact of physical exercise on non-cognitive ability in different groups. The influence of physical exercise on social behavior and educational expectation is consistent across different genders, household registration types, only-child status, boarding situations and family economic groups. Therefore, social behavior and educational expectations are not shown in the table. Boarding students have advantages over non-boarding students in developing communication ability through physical exercise (*p* < 0.001). Students from an agricultural household have more advantages in developing their communication ability through physical exercise than those from non-agricultural households (*p* < 0.05). In terms of the ability of physical exercise to promote school adaptation, girls have more advantages than boys, and those who are not an only child have more advantages than those who are an only child (*p* < 0.05). When physical exercise promotes the development of creative thinking, boarding students have more advantages than non-boarding students (*p* < 0.05). Taken together, we should fully consider the group differences in physical exercise in promoting the development of non-cognitive ability, so as to provide a foundation for students’ personal development.

## 4. Discussion

The results show that, after controlling for the influence of other factors, physical exercise has a promoting effect on six non-cognitive abilities—social behavior, communication ability, perseverance, school adaptation, educational expectation and creative thinking—and there is no significant effect on emotional control. Further, the development of non-cognitive ability in physical exercise shows certain group differences. Boarding students are the dominant group in the development of communication ability in physical exercise, agricultural household students are the dominant group in the development of perseverance in physical exercise, boys and those who are the only child are the dominant groups in the development of school adaption in physical exercise, and boarding students are the dominant group in the development of creative thinking in physical exercise.

The results of this study show that physical exercise can promote the standardization of adolescent social behavior. However, there are two opposite research results on the social behavior consequences of teenagers’ participation in physical exercise, positive and negative, and the difference mainly depends on the degree of physical exercise norms [30]. Studies have shown that actively guiding teenagers to participate in physical exercise based on the principles of rules, fairness, cooperation and moderation is conducive to reducing the incidence of deviant social behaviors [31,32]. Physical exercise can effectively prevent and reduce the harmfulness of anti-social behaviors among marginalized adolescents [33]. Physical exercise can also significantly improve the level of pro-social behavior of rural left-behind children [34]. This may be related to the value attribute of sports itself to develop teenagers’ moral judgment and cultivate their awareness of rules [35]. 

This study shows that physical exercise can promote the communication ability of junior high school students. Physical exercise provides an interactive platform for teenagers to improve their social communication ability. Physical exercise is an important means for boarding students to develop their communication skills, which may be because physical exercise improves the frequency and effect of interpersonal communication of boarding students. Participation in physical exercise can promote the formation of interpersonal relationships and increase popularity, facilitate peer acceptance, enhance friendships and promote the generation of individual social capital [36]. The interpersonal skills of teenagers who participate in moderate or above sports activities are significantly higher than those who engage in small amounts of physical exercise [37,38,39]. Within a moderate range, college students’ participation in physical exercise is positively correlated with their interpersonal communication ability, and participation in collective physical exercise projects has a more obvious effect on improving their interpersonal communication ability [40]. The influence of the type, intensity, frequency and time of physical exercise on the non-cognitive ability of junior high school students needs to be further explored in future research. 

We found that physical exercise can promote the development of school adaptation. In terms of school adaptation, the academic circle focuses on sports participation and discusses the integration of migrants into cities [39,41]. There are few studies on the relationship between physical exercise and school adaptation. This study focuses on the effect of teenagers’ participation in physical exercise on their integration into collective school life. Good collective integration is an important guarantee for teenagers to adapt to school life. Appropriate physical exercise can improve the school adaptability of teenagers [42], which is conducive to physical and mental health development. The effect of physical exercise on school adjustment was better among boys and only children, which may be related to physiological differences and the home environment. 

According to the research results of this paper, physical exercise and learning perseverance present a significant positive correlation. Learning perseverance and academic performance are two different but highly related concepts. Good learning perseverance is an important guarantee for excellent academic performance. The mechanism of sports promoting cognitive ability and academic performance has been confirmed in terms of neurophysiological, cognitive and psychological mechanisms [43,44]. An endurance sports program is an effective way to cultivate adolescent perseverance [45,46]. Agricultural household’ students are more likely to benefit from physical exercise, which can improve their learning perseverance. This provides a new perspective for improving the level of rural education.

This study found that physical activity enhances educational expectations among adolescents. Some studies have found that people with high academic qualifications also have a high incidence of physical exercise. Is this phenomenon a group characteristic or a certain causal relationship? Overall, at the same time, there are studies that have found that physical exercise in high school can lead to a 4.2–14.8% increase in wages [12]. Moreover, scholars, using data from a Canadian population survey, have found that physical exercise can lead to a 10–20% increase in incomes for individuals [47]. Return on education is an important topic in economics and has been validated by numerous investigators worldwide [48]. By studying the impact of physical exercise on educational expectations, this paper provides a new idea for exploring the relationship between physical exercise and higher education, and between physical exercise and personal income premium.

The research results in this paper show that physical exercise cannot significantly improve emotional control ability, which may be because the relationship between physical exercise and emotional control is affected to a certain extent by exercise intensity [49] and exercise items [50], etc., while the paper is limited by indexes provided by the CEPS (2014–2015) database. We were unable to obtain more detailed physical exercise information, resulting in the inability to reproduce previous research results, which needs to be further explored in subsequent studies.

Physical exercise can develop the potential of the brain and play a positive role in promoting creative thinking ability [51,52]. Aerobic exercise [53], fast walking [54] and other sports can significantly improve students’ creativity and have an important impact on their academic performance. All these are consistent with the research results of this paper, whereby physical exercise can promote creative thinking. However, in a systematic review, it was found that 77% were vulnerable to a moderate–high risk for methodological bias, suggesting that adherence to standardized and controlled research initiatives should be promoted [55].

This study has both theoretical and practical implications. In theory, this study complements the literature that investigates physical exercise in terms of promoting the development of teenagers’ non-cognitive ability. The results support the possibility that physical exercise is an important means to develop non-cognitive ability. We hope that our research can provide a reference for improving the quality of youth education. In a practical sense, physical exercise can improve the non-cognitive ability of adolescents and play a positive role in shaping a healthy personality. Therefore, schools, families and communities should jointly promote physical activity among adolescents.

Lastly, it should be pointed out that there are still some deficiencies in this study. Firstly, the data of the CEPS do not distinguish the duration of physical exercise in different sports, so they cannot reveal differences in the effects of physical exercise in different sports on teenagers’ non-cognitive abilities. Secondly, due to the limitations of the research data, this study failed to examine the mechanisms behind physical exercise’s effects on non-cognitive abilities. Finally, the research method adopted in this study is not an experimental research method. In future studies, researchers could collect information about the time, intensity and different types of exercise, and explore the impacts of different times, intensities and types of exercise on the development of adolescents’ non-cognitive abilities. In order to compensate for the shortcomings of the research methods, future research on physical exercise and the development of non-cognitive ability could benefit from the research methods of personality neuroscience and behavioral genetics to measure non-cognitive ability more objectively, so as to improve the research validity.

## 5. Conclusions

It is generally believed that the function of physical exercise is only to strengthen the body. However, our research found that physical exercise can improve the social behavior, communication ability, perseverance, school adaptation, educational expectation and creative thinking of adolescents. Physical exercise has a good adaptability in promoting the non-cognitive ability of different groups. These non-cognitive abilities are of great significance to the future achievements of adolescents.

## Figures and Tables

**Table 1:** Demographic information, physical exercise and non-cognitive ability scores of the selected subjects.

Variables	All (*n* = 7904)	Boys (*n* = 4030)	Girls (*n* = 3874)	*p*-Value
***Dependent variables***				
Social behavior	3.35 (1.01)	3.12 (1.09)	3.58 (0.86)	<0.001
Communication ability	63.88 (22.27)	60.72 (23.49)	67.14 (20.42)	<0.001
Perseverance	72.04 (25.17)	69.68 (25.98)	74.49 (24.06)	<0.001
Adaptation ability	68.65 (23.03)	67.29 (23.77)	70.08 (22.14)	<0.001
Educational expectation	16.41 (3.25)	16.12 (3.49)	16.69 (2.96)	<0.001
Emotional control	29.42 (21.09)	28.54 (21.93)	30.35 (20.13)	<0.001
Creative thinking	74.00 (20.84)	73.59 (21.43)	74.42 (20.21)	0.081
***Independent variable***				
**Physical exercise** **(** **h** **)**	0.37 (0.41)	0.43 (0.49)	0.30 (0.28)	0.152
***Covariates***				
Parent–child relationship	2.61 (0.46)	2.60 (0.47)	2.62 (0.45)	0.033
Cognitive ability	0.34 (0.81)	0.31 (0.84)	0.37 (0.77)	<0.01
Academic score	65.56 (19.07)	61.60 (20.05)	69.69 (17.04)	<0.001
Personality traits	31.53 (23.90)	31.24 (24.73)	31.82 (23.01)	0.283
Peer group	2.28 (0.67)	2.08 (0.71)	2.47 (0.55)	<0.001
Teachers’ attention	55.10 (23.62)	54.35 (24.67)	55.88 (22.45)	<0.001
Family education capital	10.94 (3.05)	10.89 (3.07)	10.99 (3.02)	<0.01
Household registration type, *n* (%)				
Agricultural household	4149 (52.49)	2159 (53.60)	1990 (51.40)	0.050
Non-agricultural household	3755 (47.51)	1871 (46.40)	1884 (48.60)	
Only-child status, *n* (%)				
Only child	3573 (45.20)	1954 (48.49)	1619 (41.79)	<0.001
Not only child	4331 (54.80)	2076 (51.51)	2255 (58.21)	
Preschool experiences, *n* (%)				
yes	6504 (82.29)	3303 (81.96)	3201 (82.63)	0.437
no	1400 (17.71)	727 (18.04)	673 (17.37)	
Boarding situations, *n* (%)				
yes	2401 (30.38)	1227 (30.45)	1174 (30.30)	0.891
no	5503 (69.62)	2803 (69.55)	2700 (69.70)	
After-school interest class, *n* (%)				
yes	3877 (49.05)	2129 (52.83)	1748 (45.12)	<0.001
no	4027 (50.95)	1901 (47.17)	2126 (54.88)	
Parental relationship, *n* (%)				
good	6814 (86.21)	3506 (87.00)	3308 (85.39)	0.038
bad	1090 (13.79)	524 (13.00)	566 (14.61)	
Health condition, *n* (%)				
good	5096 (64.47)	2686 (66.65)	2410 (62.21)	<0.001
bad	2808 (35.53)	1344 (33.35)	1464 (37.79)	
Family economy, *n* (%)				
enrichment	521 (6.59)	266 (6.60)	255 (6.58)	0.974
poverty	7383 (93.41)	3764 (93.40)	3619 (93.42)	
Parents’ company, *n* (%)				
good	6131 (77.57)	3142 (77.97)	2989 (77.16)	0.388
bad	1773 (22.43)	888 (22.03)	885 (22.84)	

Data were described as *n* (%) or mean ± SD.

**Table 2:** Regression analysis of the influence of physical exercise on non-cognitive ability.

	Social Behavior	Communication Ability	Perseverance	School Adaptation	Educational Expectation	Emotion Control	Creative Thinking
Physical exercise	0.119 ***	1.935 **	1.777 *	4.420 ***	0.251 **	−0.106	1.753 **
	(0.025)	(0.614)	(0.692)	(0.589)	(0.087)	(0.584)	(0.573)
Gender	−0.255 ***	−4.064 ***	−0.509	−0.955	0.126	−2.521 ***	1.380 **
	(0.021)	(0.522)	(0.588)	(0.501)	(0.073)	(0.491)	(0.486)
Household registration type	−0.020	−0.676	0.276	0.027	−0.128	−0.581	−1.332 *
	(0.024)	(0.583)	(0.656)	(0.559)	(0.082)	(0.547)	(0.543)
Only-child status	0.048 *	1.274 *	−0.599	0.160	−0.138	−1.185 *	2.707 ***
	(0.023)	(0.555)	(0.625)	(0.532)	(0.078)	(0.522)	(0.517)
Preschool experiences	0.030	1.257	1.046	0.116	−0.077	−0.250	−0.780
	(0.026)	(0.644)	(0.724)	(0.618)	(0.090)	(0.606)	(0.601)
Boarding situations	−0.060 *	1.649 **	−2.030 **	−1.073	−0.144	0.731	−1.735 **
	(0.024)	(0.598)	(0.673)	(0.574)	(0.083)	(0.563)	(0.557)
After-school interest class	−0.050 *	−1.313 *	0.042	−2.422 ***	−0.300 ***	−1.590 **	−1.273 **
	(0.022)	(0.528)	(0.595)	(0.507)	(0.074)	(0.497)	(0.492)
Parental relationship	0.064 *	−0.875	2.715 **	1.511 *	−0.005	−5.603 ***	0.016
	(0.031)	(0.744)	(0.838)	(0.715)	(0.105)	(0.700)	(0.692)
Cognitive ability	0.070 ***	0.689	0.096	0.003	0.426 ***	−0.709	1.777 ***
	(0.016)	(0.393)	(0.441)	(0.377)	(0.055)	(0.369)	(0.365)
Academic score	0.004 ***	0.036 *	0.192 ***	0.042 *	0.048 ***	0.012	0.118 ***
	(0.001)	(0.018)	(0.020)	(0.017)	(0.002)	(0.017)	(0.016)
Personality traits	−0.001 ***	0.019	0.019	−0.122 ***	0.002	0.205 ***	−0.038 ***
	(0.000)	(0.010)	(0.012)	(0.010)	(0.001)	(0.010)	(0.010)
Peer group	0.474 ***	4.968 ***	7.005 ***	4.583 ***	0.706 ***	−3.956 ***	2.908 ***
	(0.017)	(0.420)	(0.472)	(0.403)	(0.059)	(0.395)	(0.392)
Teachers’ attention	0.005 ***	0.141 ***	0.175 ***	0.302 ***	0.009 ***	−0.004	0.094 ***
	(0.000)	(0.011)	(0.012)	(0.010)	(0.002)	(0.010)	(0.010)
Family education capital	0.006	0.133	−0.224 *	0.002	0.098 ***	0.206 *	0.563 ***
	(0.04)	(0.096)	(0.108)	(0.092)	(0.014)	(0.091)	(0.090)
Family economic	0.000	−0.899	0.108	2.440 **	0.087	−2.502 **	4.133 ***
	(0.040)	(0.973)	(1.095)	(0.933)	(0.136)	(0.915)	(0.899)
Health condition	0.139 ***	3.094 ***	3.733 ***	4.435 ***	0.058	−6.703 ***	2.457 ***
	(0.021)	(0.525)	(0.591)	(0.504)	(0.073)	(0.493)	(0.488)
Parent–child relationship	0.219 ***	7.468 ***	2.376 ***	5.498 ***	0.377 ***	−5.499 ***	3.861 ***
	(0.024)	(0.581)	(0.653)	(0.558)	(0.082)	(0.546)	(0.541)
Parents’ company	−0.035	−0.595	−0.204	−1.172 *	0.238 **	2.522 ***	−0.244
	(0.025)	(0.613)	(0.690)	(0.589)	(0.086)	(0.578)	(0.570)
_cons	1.087 ***	20.780 ***	25.040 ***	24.500 ***	8.930 ***	55.230 ***	36.440 ***
	(0.0910)	(2.230)	(2.510)	(2.141)	(0.314)	(2.100)	(2.074)
R2	0.306	0.153	0.154	0.275	0.259	0.183	0.177

Standard errors in parentheses; * *p* < 0.05, ** *p* < 0.01, *** *p* < 0.001.

**Table 3:** Group differences in the influence of physical exercise on non-cognitive ability.

Interactive Item	CA	Pe	SA	CT
Gender × PE (Reference: girl)				
boy	−0.410	−2.779	−4.029 **	−1.246
Household registration type × PE (Reference: Non-agricultural household)				
Agricultural household	2.251	2.826 *	0.853	−0.638
Only-child status × PE (Reference: Not only child)				
Only child	0.955	−1.299	−2.251 *	0.183
Boarding situation × PE (Reference: no)				
yes	5.565 ***	1.357	0.351	3.340 *
Family economic status × PE (Reference: poverty)				
enrichment	−1.768	−2.705	2.488	1.440

CA, communication ability; Pe, perseverance; SA, school adaptation; CT, creative thinking; PE, physical exercise; * *p* < 0.05, ** *p* < 0.01, *** *p* < 0.001.

## Data Availability

The China Educational Panel Survey (CEPS) dataset is publicly available at http://www.cnsda.org/index.php?r=projects/view&id=61662993 (accessed on 26 June 2020).
